# Lactoferrin and Its Enzymatic Hydrolysates as Natural Antimicrobial and Antioxidant Agents for Food Preservation

**DOI:** 10.3390/foods15061052

**Published:** 2026-03-17

**Authors:** Špela Gruden, Petra Mohar Lorbeg, Bojana Bogovič Matijašić, Mihaela Skrt, Adrijana Leonardi, Igor Križaj, Nataša Poklar Ulrih

**Affiliations:** 1 Department of Food Science and Technology, Biotechnical Faculty, University of Ljubljana, Jaminkarjeva 101, 1000 Ljubljana, Slovenia; spela.gruden@mf.uni-lj.si (Š.G.); mihaela.skrt@bf.uni-lj.si (M.S.); 2 Institute of Biochemistry and Molecular Genetics, Faculty of Medicine, Universety of Ljubljana, Vrazov trg 2, 1000 Ljubljana, Slovenia; 3 Department of Animal Science, Biotechnical Faculty, University of Ljubljana, Groblje 3, 1230 Domžale, Slovenia; petra.moharlorbeg@bf.uni-lj.si (P.M.L.); bojana.bogovicmatijasic@bf.uni-lj.si (B.B.M.); 4 Department of Molecular and Biomedical Sciences, Jožef Stefan Institute, Jamova cesta 39, 1000 Ljubljana, Slovenia; adrijana.leonardi@ijs.si (A.L.); igor.krizaj@ijs.si (I.K.)

**Keywords:** lactoferrin, lactoferrin peptides, enzymatic hydrolysis, antibacterial activity, antioxidant activity, food preservation

## Abstract

Lactoferrin (Lf) and Lf-derived peptides are multifunctional milk components with potential applications in food preservation due to their antibacterial and antioxidant properties. In this study, the antibacterial and antioxidant activities of bovine lactoferrin and Lf-derived peptides obtained by enzymatic hydrolysis with pepsin, trypsin, and chymotrypsin were evaluated. Antibacterial activity was assessed against four foodborne pathogens and spoilage microorganisms (*Escherichia coli*, *Listeria monocytogenes*, *Staphylococcus epidermidis*, and *Latilactobacillus sakei*), while antioxidant activity was determined using four complementary assays. Lf showed stronger antibacterial activity than the corresponding hydrolysates against all tested strains, while the hydrolysates notably inhibited *Listeria monocytogenes* and *Latilactobacillus sakei*. Both Lf and its peptides showed lower antioxidant capacity than Trolox, although native Lf and its peptides markedly inhibited lipid peroxidation. Lf peptides demonstrated greater antioxidant activity in the superoxide scavenging and FRAP assays. Low-molecular-weight peptides (<10 kDa) contributed most to antioxidant activity, while mass spectrometry analysis revealed peptide sequences rich in hydrophobic and electron-donating amino acid residues, providing mechanistic insight into the observed activities. Overall, these findings highlight the potential of lactoferrin and its enzymatic hydrolysates as natural antimicrobial and antioxidant agents for food preservation.

## 1. Introduction

Milk proteins play an important role in the food industry as a source of high nutritional value and as functional ingredients that influence the texture, stability, and overall quality of food products due to their diverse physicochemical and functional properties [1]. Beyond intact proteins, increasing attention has been directed towards their hydrolysis products, particularly bioactive peptides, due to their potential technological and biological functions in food systems [2]. Among dairy proteins, lactoferrin (Lf) is one of the most extensively studied, owing to its broad spectrum of biological activities, including antimicrobial (antibacterial, antiviral, antifungal, and antiparasitic), antitumour, anti-inflammatory, immunomodulatory, and antioxidant effects [3]. Lactoferrin is a 78 kDa iron-binding glycoprotein synthesised by epithelial cells in various organs and secreted into several exocrine fluids, with the highest concentrations found in colostrum and milk [4].

Due to Lf’s multifunctional properties and important role in human health [5], Lf is widely incorporated into food supplements and infant formulas [6,7]. Both the U.S. Food and Drug Administration (FDA) [5] and the European Food Safety Authority (EFSA) [7] recognise Lf as a safe ingredient for food applications. Lf also shows great potential for use in the dairy and meat industries as a natural additive capable of delaying spoilage and extending product shelf life [6] due to its antimicrobial and antioxidant activities. Food preservation is essential for maintaining food quality and safety, as foods are highly susceptible to deterioration caused by microbial, chemical, and physical processes. Microbial contamination, primarily by bacteria, yeasts, and moulds, is a major cause of food spoilage and foodborne illness, leading to undesirable changes in texture, odour, appearance, and nutritional value [8]. Oxidative processes are another major contributor to food degradation. Free radicals damage essential components such as lipids and proteins, compromising food quality and stability. Lipids, which are key constituents of many foods and biological systems, are particularly vulnerable to oxidation when exposed to light or elevated temperatures [9]. Lipid oxidation generates reactive species that further promote protein oxidation and structural degradation [10], ultimately resulting in losses of nutritional value, flavour, texture, and appearance, and shortening shelf life. Antioxidants, whether natural or synthetic, play a crucial role in preventing oxidative spoilage manifested as lipid rancidity, discolouration, nutrient loss, and reduced sensory quality [11]. Moreover, natural antioxidants incorporated into functional foods may also confer health benefits by mitigating oxidative stress [12].

Lf is well recognised for its antimicrobial activity against a broad range of Gram-positive and Gram-negative bacteria [13], including common foodborne pathogens and spoilage microorganisms [14,15,16]. Importantly, Lf retains its antibacterial activity following pasteurisation [17] and under processing conditions relevant to meat products [18]. In addition to native Lf, antibacterial properties have also been reported for Lf-derived peptides generated through enzymatic hydrolysis or designed based on the structural characteristics of antimicrobial peptides. Over the past three decades, the antibacterial activity of Lf-derived peptides has been extensively investigated [19,20,21,22,23]. While native Lf exhibits substantial antimicrobial activity, its digestion products, such as lactoferricin (Lf-cin), often display enhanced potency, highlighting their potential as natural antimicrobial agents for human and veterinary applications [24,25]. Beyond medical use, these peptides also represent promising candidates for food preservation strategies, offering a natural approach to improving food safety and shelf life [26,27].

In addition to its antimicrobial properties, the antioxidant activity of Lf represents another important function that can be exploited for food preservation. The antioxidant activity of Lf is primarily linked to its ability to bind iron, an essential element for cellular growth that can promote oxidative reactions when present in excess [28]. Compared with its antimicrobial properties, the antioxidant activity of Lf has received less attention. Studies investigating the antioxidant effects of Lf have been conducted in vivo [29,30] and in vitro [31,32,33]. In addition to native proteins, bioactive peptides derived from food proteins have been widely reported to exhibit antioxidant activity, with effectiveness influenced by peptide size and amino acid composition [34]. Similar antioxidant properties have been demonstrated for lactoferrin digestion products [32,33].

The aim of the present study was to evaluate the antibacterial and antioxidant activities of Lf and Lf-derived peptides obtained by enzymatic hydrolysis using three different proteases. In addition to pepsin, whose digestion of Lf is well known to generate antibacterial peptides, trypsin and chymotrypsin were also employed, as they have been less frequently investigated for Lf hydrolysis. While most previous studies have focused on the biological activity of individual, isolated Lf-derived peptides, comparatively few investigations have examined whole Lf hydrolysates, despite their greater feasibility and cost-effectiveness for industrial-scale production. Antibacterial activity was assessed against four bacterial strains (*Escherichia coli*, *Listeria monocytogenes*, *Staphylococcus epidermidis*, and *Latilactobacillus sakei*) representing foodborne pathogens and spoilage microorganisms commonly found in meat and dairy products. Antioxidant activity was evaluated using four complementary assays to assess activity against different types of reactive species. Furthermore, hydrolysates were fractionated into high- (10–50 kDa) and low-molecular-weight (<10 kDa) peptide fractions, and mass spectrometry analysis was performed to characterise peptide profiles and to gain insight into the mechanisms underlying the observed biological activities.

## 2. Materials and Methods

### 2.1. Materials

Lactoferrin was isolated from acid whey as part of the project LAKTIKA (OP20.03521) with the use of ion-exchange chromatography on CIM^®^ monolithic columns [35]. Pepsin from porcine gastric mucosa, trypsin and α-chymotrypsin from bovine pancreas were purchased from Sigma-Aldrich (St. Louis, MO, USA). The following chemicals were also purchased from Sigma-Aldrich: calcium chloride (CaCl_2_), trypsin inhibitor from *Glycin max*, sodium dodecyl sulphate (SDS), acetonitrile, trichloroacetic acid (TCA), 2,2-diphenyl-1-picrylhydrazyl (DPPH), Trolox, Tris-HCl, pyrogallol, Na-acetate, 2,4,6-Tris(2-pyridyl)-s-triazine (TPTZ), iron (III) chloride hexahydrate (FeCl_3_ × 6H_2_O), ferrous sulphate heptahydrate (FeSO_4_ × 7 H_2_O), phosphate buffer, linoleic acid and ammonium thiocyanate. Sodium hydroxide (NaOH) and hydrochloric acid (HCl) were purchased from Merck (Darmstadt, Germany) while ethylenediaminetetraacetic acid (EDTA) was purchased from Kemika (Zagreb, Croatia).

### 2.2. Enzymatic Hydrolysis of Lactoferrin with Pepsin, Trypsin, and Chymotrypsin

The enzymatic hydrolysis of Lf was performed using pepsin, trypsin, and chymotrypsin, following the method of Recio and Visser (1999) [23], and Lizzi et al. (2016) [21], with some modifications. A 5% (*w*/*v*) Lf solution was prepared with ultrapure water (Milli-Q water). For hydrolysis with pepsin, the pH of the Lf solution was adjusted to 3.0 by gradually adding 1 M HCl. For hydrolysis with trypsin and chymotrypsin, the pH was adjusted to 8.0 by gradually adding 1 M NaOH. Subsequently, 1 mL of a 3% pepsin or 6% trypsin and chymotrypsin aqueous solution (*w*/*w* based on the mass of Lf used) was added to the Lf solution. For trypsin/chymotrypsin hydrolysis, 1 mL of 2 M CaCl_2_ (final molar concentration 0.02 M) was also added before the addition of the enzymes. The solution was incubated for 4 h at 37 °C in a water bath. The pepsin reaction was stopped by gradually increasing the pH to 8.0 with 1 M NaOH, while the trypsin/chymotrypsin reactions were inhibited by adding 1 mL of trypsin inhibitor, according to the manufacturer’s instructions. The Lf hydrolysates were then lyophilised.

### 2.3. Separation of Lactoferrin Peptides to Obtain Lf Peptides with Different Molecular Weights

Lf hydrolysates, obtained as described above, were separated by size (kDa) using centrifugal filters (Amicon^®^ Ultra 15 mL, Merck Millipore, Burlington, MA, USA) with molecular weight cut-offs (MWCO) of 50 kDa and 10 kDa. Centrifugation was performed using a centrifuge (Rotanta 460-R, Hettich, Beverly, MA, USA)) for 10 min at 20 °C and 5000× *g*. First, filters with a 50 kDa MWCO were used to obtain peptides smaller than 50 kDa. The process was then repeated with a 10 kDa MWCO filter to obtain peptides smaller than 10 kDa and peptides with a molecular weight of 10–50 kDa.

### 2.4. Determination of Peptide Sequence by Mass Spectrometry

The dry hydrolysates of Lf were weighed and dissolved in water at a concentration of 1 µg/µL. 5 µg of each hydrolysate solution was reduced with 10 mM tris(2-carboxyethyl)phosphine and alkylated with 40 mM chloroacetamide in 30 µL of 50 mM NH_4_HCO_3_ containing 6.0 M urea in the dark at room temperature for 30 min. The reaction mixtures were then diluted to 250 µL with 25 mM NH_4_HCO_3_ and 60 ng of peptides were analysed by mass spectrometry as previously described [36].

The tandem mass spectra were exported as generic Mascot files (MGF) and searched by the Mascot software v3.0 (Matrix Science Ltd., London, UK) against the bovine lactotransferrin sequence with UniProt accession number P24627 (TRFL_BOVIN) using the following parameters: 20 ppm peptide and 0.6 Da fragment mass error tolerance, 2 enzyme missed cleavages, 2+ and 3+ peptide charges, carbamidomethyl-Cys as fixed, oxidised Met and deamidated Asn and Gln as variable modifications, and an automated Decoy database search. Scaffold software v5.3.3 (Proteome Software, Portland, OR, USA) with a target false discovery rate for peptides of 1% was used to validate the Mascot results.

### 2.5. Determination of Antimicrobial Activity (MIC) of Lactoferrin and Lactoferrin Derived Peptides by Microdilution Assay

Minimal inhibitory concentration (MIC) of Lf and Lf-derived peptides was assessed by microdilution test in 96-well plates (Brand GmbH, Germany), using the following bacterial strains: *Escherichia (E.) coli* O157: H7 tox- IM219 (ZIM-Culture Collection of Industrial Microorganisms, Ljubljana, Slovenia, registered as WDCM810), *Latilactobacillus (Lb.) sakei* ATCC 15522 (American Type Culture Collection, USA), *Listeria (Li.) monocytogenes* IM221 (ZIM) and *Staphylococcus (S.) epidermidis* ATCC 14990.

Two-fold consecutive dilutions of Lf in water were applied to microtiter plates so that the final concentration of Lf was from 19.25 to 0.019 mg/mL for all test strains except *Lb. sakei*, or 2.4 to 0.0025 mg/mL when *Lb. sakei* was used. Moreover, Lf hydrolysate and peptides derived from the above-mentioned concentrations of Lf were also applied to microtiter plates. The last column in the microtiter plate contained only diluted bacterial strains and served as a positive control. Bacterial strains were grown on the surface of brain-heart agar (Merck, Darmstadt, Germany) at 37 °C for 18 h, and *Lb. sakei* was grown on MRS agar (Merck, Darmstadt, Germany) at 30 °C for 18 h in anaerobic conditions. The colonies were resuspended in ¼ strength Ringer solution (Merck, Darmstadt, Germany) to reach turbidity according to McFarland 1 (OD_600_ 0.123 ± 0.005). These suspensions were diluted 250-times in two-fold brain–heart broth or two-fold MRS broth (*Lb. sakei*) and applied (50 µL) into each well, which already contained 50 µL of diluted samples. The plates were incubated at 37 °C for 6 h (plates with *E. coli* strain) or 18 h (plates with *Li. monocytogenes* and *S. epidermidis*), while the plates with *Lb. sakei* were incubated for 20 h at 30 °C under anaerobic conditions. After incubation, OD was measured at 630 nm (BIO-TEK EL 808; BioTek Instruments, Winooski, VT, USA). The OD_630_ of the uninoculated medium with diluted samples was subtracted from the measured OD_630_ values for each individual sample. MIC was defined as the minimal concentration that inhibited the growth of the indicator bacteria by at least 50%. All assays were performed in duplicate, and the tests were repeated three times.

### 2.6. Determination of Antioxidant Activity of Lactoferrin and Lactoferrin-Derived Peptides

#### 2.6.1. DPPH Radical Scavenging Activity

The DPPH radical scavenging activity of Lf and Lf peptides was measured using a spectrophotometer (BioSpectrometer basic) at 517 nm. The DPPH stock solution (5 mM) was prepared in 100% (*v*/*v*) methanol and thoroughly dissolved by stirring for 2 h in the dark at room temperature. A working solution of 0.1 mM DPPH in 50% (*v*/*v*) methanol was prepared from the stock solution of DPPH. Lyophilised Lf and Lf peptides were dissolved in dH_2_O at a concentration of 5 mg/mL. Samples were prepared by adding 50 μL of Lf or Lf peptide solution to 950 μL of DPPH (0.1 mM in 50% methanol). The final concentration of Lf or Lf peptides was 0.25 mg/mL. The blank contained 950 μL methanol (50% *v*/*v*) and 50 μL dH_2_O. The positive control, Trolox, was prepared by adding 50 μL of Trolox to 950 μL of DPPH (0.1 mM) at the same concentration as the tested samples (c = 0.25 mg/mL). All measurements were performed in triplicate. Antioxidant activity was monitored for 4 h, and the samples were kept in the dark during the measurement. The measured absorbance values of the samples at 517 nm were converted into DPPH scavenging activity (%) using the following equation, where A is the absorbance of the sample or negative control:

(1) $$DPPH\;scavenging\;activity\left(\%\right)={[(A}_{neg.control}-\;A_{sample})/A_{neg.control}]\times \;100$$

#### 2.6.2. Superoxide Radical Scavenging Activity (O_2_^• −^)

Superoxide scavenging activity of Lf and Lf peptides was performed based on the previously described method with minor modifications [37]. Lf and Lf peptides were dissolved in 50 mM Tris buffer (pH 7.4) with 1 mM EDTA at a concentration of 0.375 mg/mL. Pyrogallol (1.5 mM) was prepared in 10 mM HCl. Superoxide scavenging activity measurements were performed on a Safire 2 microtiter plate reader (Tekan) at 420 nm. 80 μL of sample and 40 μL of pyrogallol were mixed in each well of the microtiter plate. Tris buffer with EDTA was used as a blank. As a positive control, we used Trolox prepared according to the same procedure as the other analysed samples. The final concentration of Lf and Lf peptides in the microtiter plate wells was 0.25 mg/mL. Superoxide scavenging activity was measured every 30 min for a total of 4 h. All measurements of inhibition of superoxide radicals were performed in triplicate. The obtained values were converted into superoxide scavenging activity (%) according to Equation (2), where A is the absorbance of the sample or the negative control.

(2) $$Superoxide\;scavenging\;activity\left(\%\right)={[(A}_{neg.control}-A_{sample})/A_{neg.control}]\times 100$$

#### 2.6.3. Ferric Reducing Antioxidant Power (FRAP) Assay

The FRAP assay of Lf and Lf peptides was measured in the presence of 2,4,6-Tris(2-pyridyl)-s-triazine (TPTZ) reagent according to the method of Liu et al. (2019) with minor adjustments [38]. First, the FRAP reagent and the stock solutions of Lf and Lf peptides were prepared in milliQ water (5 mg/mL). FRAP reagent was prepared by mixing 0.3 M Na-acetate (pH 3.6), 10 mM TPTZ in 40 mM HCl and 20 mM FeCl_3_ · 6H_2_O at a ratio of 10:1:1 (*v*/*v*/*v*). FRAP measurements were performed using a Safire 2 microtiter plate reader (Tekan) at 593 nm. Samples were prepared in microtiter plates. To each well, 20 μL of Lf or Lf peptides and 150 μL of FRAP reagent were added. The final concentration of Lf and Lf peptides in each well was 0.25 mg/mL. A positive control, Trolox, was prepared following the same procedure as the samples. FRAP antioxidant activity was measured as a function of time, after 15 min, 30 min, 1 h, 2 h and 4 h. Obtained absorbances of Lf and Lf peptides at 593 nm were compared to the standard calibration curve. The standard was prepared by dissolving FeSO_4_ · 7H_2_O in milliQ water at concentrations ranging from 10 to 500 μM. The measurements of the samples and the standard were performed in three parallel runs. The results are expressed as μmol Fe^2+^, reduced to the mass (mg) of the protein.

#### 2.6.4. Inhibition of Lipid Peroxidation

The lipid peroxidation inhibition activity of Lf and Lf peptides was measured according to the method of Chi et al. (2015) with minor modifications [39]. Lf and Lf peptides were dissolved in 10 mL phosphate buffer (50 mM, pH 7.0) to a concentration of 0.625 mg/mL. To the Lf and Lf peptide solution, 10 mL of ethanol (100%) and 130 μL of linoleic acid were added and made up to the 25 mL mark with milliQ water. The positive control was prepared according to the same procedure as the samples and contained Trolox instead of protein or peptides. The samples were incubated at 40 °C in a water bath for 7 days. On each day of measurement, 100 μL Lf or Lf peptides with linoleic acid were mixed with 4.7 mL ethanol (75% *v*/*v*), 100 μL ammonium thiocyanate (30% *w*/*v*), and 100 μL FeCl_3_ (20 mM in 3.5% (*v*/*v*) HCl). The reaction mixture was thoroughly mixed using a vortex mixer and left to stand at room temperature for 3 min. Afterwards, 400 μL of ethanol (75% *v*/*v*) and 400 μL of the reaction mixture were added into a plastic cuvette, and the absorbance was measured at 500 nm using a spectrophotometer (Eppendorf BioSpectrometer basic, Hamburg, Germany). The degree of oxidation of the linoleic acid is proportional to the absorbance values. All measurements were performed in triplicate.

## 3. Results

### 3.1. Enzymatic Hydrolysis of Lactoferrin

In this study, bovine Lf was selected for enzymatic digestion, as it is the most widely used form of Lf in the food industry. Purified bovine Lf (bLf) is traditionally isolated from milk using established biochemical separation and extraction methods. Although alternative strategies, such as recombinant expression in animals, microorganisms, and plants, are under development, these approaches are not yet commercially implemented [40]. In this context, an important aspect of the present study is the use of acid whey as the lactoferrin source [35]. Acid whey, a by-product of the dairy industry, remains largely underutilised despite its bioactive potential, so its valorisation as a source of lactoferrin represents a sustainable approach to upgrading an industrial by-product into a high-value functional ingredient.

Before the main experiments, a series of preliminary trials was conducted to evaluate the effects of pH (1.0–4.0 for pepsin and 7.0–9.0 for trypsin/chymotrypsin), reaction temperature (30–60 °C), and reaction time (1–24 h) on enzymatic hydrolysis using pepsin, trypsin, and chymotrypsin. The hydrolysis conditions described in the Methods section (Section 2.2) were selected as optimal, as several tested conditions resulted in similar peptide profiles based on HPLC analysis or showed lower antibacterial activity against *Lb. sakei* .

Several methodological adjustments to Lf hydrolysis were implemented in this study and are noteworthy. Because high temperatures can negatively affect the antibacterial activity of peptides, two approaches for terminating pepsin activity were evaluated: thermal inactivation and pH adjustment. In the first approach, the enzymatic reaction was stopped by heating the sample at 80 °C for 15 min, while in the second, the reaction was terminated by increasing the pH to 8.0, a condition under which pepsin is inactive [41]. The minimal inhibitory concentration (MIC) of pepsin-derived Lf peptides against *Lb. sakei* was 0.31 mg/mL when the reaction was terminated by heating, compared with 0.016 mg/mL when terminated by pH adjustment. As most previous studies have inactivated pepsin by heating [19,23,42], these findings indicate that pH adjustment is more appropriate in inhibiting pepsin activity while better preserving the antibacterial bioactivity of Lf-derived peptides. In addition, higher enzyme concentrations (6% *w*/*w* relative to substrate) were used for trypsin and chymotrypsin hydrolysis, as this resulted in a higher degree of Lf digestion and increased production of Lf-derived peptides (see Supplementary Figure S1).

**Figure 1:**
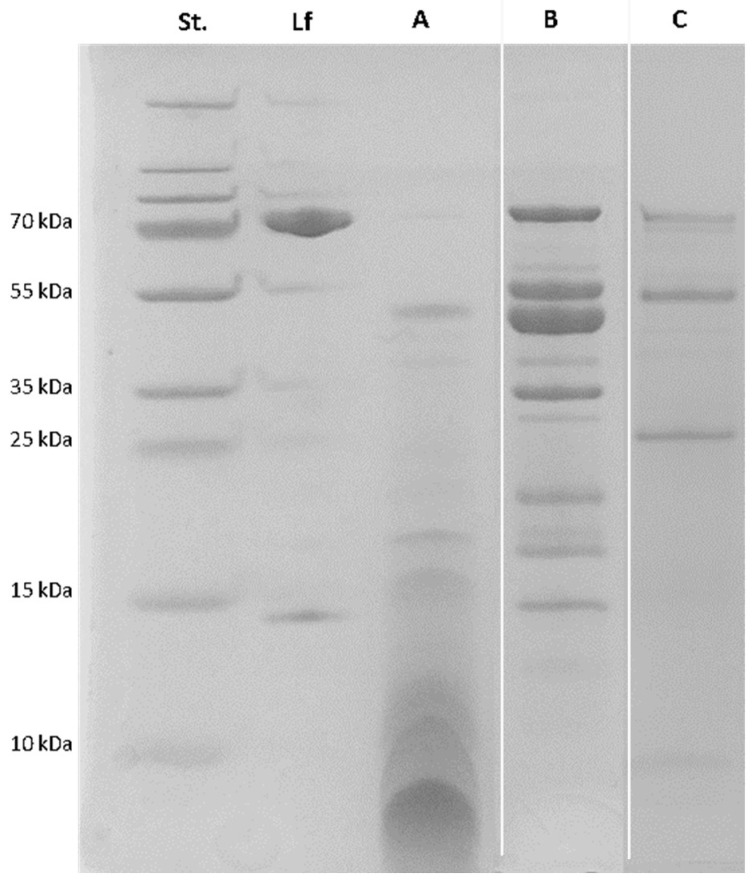
SDS-PAGE results of Lf enzymatic hydrolysis with pepsin (A), trypsin (B) and chymotrypsin (C). Samples from trypsin (B) and chymotrypsin (C) hydrolysates were electrophoresed on separate gels.

As shown in Figure 1, pepsin-mediated hydrolysis resulted in complete digestion of Lf, whereas trypsin and chymotrypsin caused only partial hydrolysis. The complete digestion observed with pepsin is likely due to the acidic conditions used during hydrolysis. At low pH (pH 3.0), Lf is denatured and therefore more susceptible to enzymatic cleavage by pepsin, whereas at alkaline pH (pH 8.0), Lf remains more structurally stable [43].

This denatured state under acidic conditions resulted mainly in the formation of smaller peptides (<15 kDa). Although partial digestion of Lf occurred when trypsin and chymotrypsin were used, it still resulted in the production of peptide fragments, mainly larger fragments, as indicated by bands between approximately 15 and 55 kDa.

### 3.2. Antibacterial Activity of Lactoferrin in Lactoferrin Peptides

The antibacterial activity of Lf and its hydrolysates, generated by pepsin, trypsin, or chymotrypsin digestion, was evaluated against four bacterial strains: *Lb. sakei*, *Li. monocytogenes*, *S. epidermidis*, and *E. coli*. As the antibacterial efficacy of antimicrobial peptides can depend on molecular weight, Lf-derived peptides were further fractionated into higher (10–50 kDa) and lower (<10 kDa) molecular weight peptides and tested separately.

As shown in Table 1, Lf inhibited the growth of all tested strains, with the strongest effect against *Lb. sakei* and the weakest against *S. epidermidis*. None of the Lf-derived peptides showed greater antibacterial activity than native Lf, most likely due to the low peptide concentration, as discussed in Section 4. Pepsin-generated hydrolysates inhibited only *Lb. sakei* and *Li. monocytogenes*, whereas trypsin- and chymotrypsin-generated hydrolysates also inhibited *E. coli*. However, this activity may be partially attributable to residual unhydrolysed Lf in these preparations, as Lf showed antibacterial effects against these strains. No inhibition was observed for *S. epidermidis*, likely reflecting the low susceptibility of this strain to native Lf. Peptide fractions in the 10–50 kDa range showed limited antibacterial activity, inhibiting only *Lb. sakei*, while peptides smaller than 10 kDa exhibited no antibacterial activity against any tested strain. Overall, these results indicate that Lf and its hydrolysates are more effective antibacterial agents than the isolated peptide fractions. Furthermore, to the best of our knowledge, this is the first report in existing literature that demonstrates the antibacterial properties of Lf and its hydrolysates against the *Lb. sakei*.

### 3.3. Antioxidant Activity of Lactoferrin and Lactoferrin Peptides

The antioxidant activity of native Lf and Lf-derived peptides was evaluated using four complementary assays targeting different reactive species and oxidation mechanisms. Trolox, a well-established antioxidant, was included as a reference to enable comparison of antioxidant potency. To specifically assess the antioxidant activity of peptides, residual non-hydrolysed Lf was removed from the hydrolysates using centrifugal filters with a molecular weight cut-off of 50 kDa; these samples are referred to as Lf peptides (<50 kDa). Antioxidant activity was also assessed for peptide fractions with molecular weights of 10–50 kDa and <10 kDa.

#### 3.3.1. DPPH Radical Scavenging Activity

**Figure 2:**
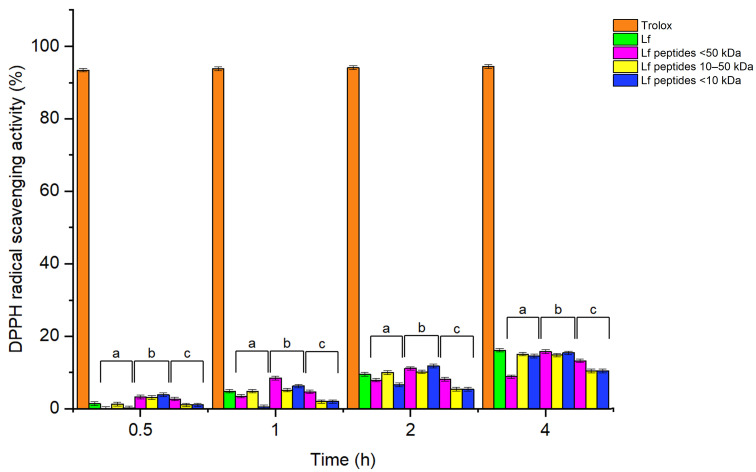
DPPH radical scavenging activity (%) of Lf and pepsin (a), trypsin (b), and chymotrypsin (c) Lf peptides as function of time (hours). The results show the DPPH radical scavenging activity of Trolox (orange), Lf (green), Lf peptides (<50 kDa), and Lf peptides with a molecular weight of 10–50 kDa and <10 kDa.

The DPPH radical scavenging activity of Lf and Lf-derived peptides is shown in Figure 2. After a 4 h assay, no major differences in DPPH radical scavenging activity were observed between Lf and Lf peptides. Both Lf and Lf peptides had lower antioxidant activity when compared to that of Trolox, which reached more than 90% DPPH radical scavenging activity after 30 min. The inhibition of DPPH● by Lf and Lf peptides was time-dependent and increased during the DPPH assay; however, it didn’t exceed 20% of DPPH● inhibition even after 4 h of assay. No significant differences in DPPH radical scavenging activity were observed between pepsin, trypsin, and chymotrypsin Lf peptides and their corresponding Lf peptides with a different molecular weight.

#### 3.3.2. Superoxide Radical Scavenging Activity

Because the superoxide anion (O_2_•^−^) plays a key role in the formation of highly reactive oxygen species such as the hydroxyl radical (OH^•^) and singlet oxygen (^1^O_2_) [44], the antioxidant activity of Lf and Lf peptides was further evaluated using a superoxide radical scavenging assay. Pyrogallol was used as a superoxide anion generator and indicator system [45].

**Figure 3:**
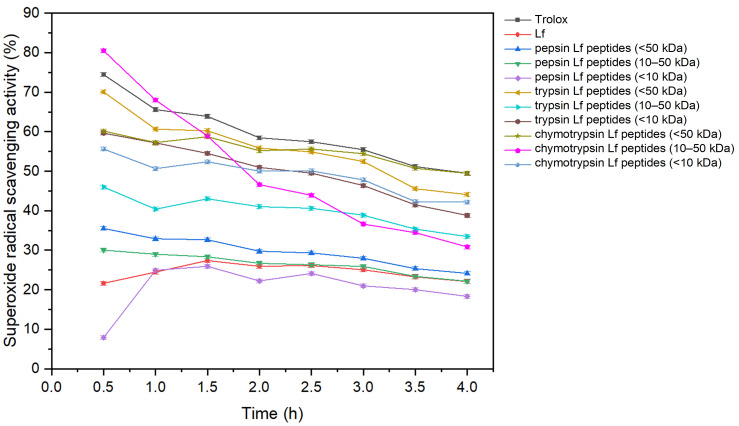
Superoxide radical scavenging activity (%) of Lf and pepsin, trypsin and chymotrypsin Lf peptides as function of time (hours). The results represent the superoxide radical scavenging activity of Trolox, Lf, Lf peptides (<50 kDa) and Lf peptides with a molecular weight of 10–50 kDa and <10 kDa.

As shown in Figure 3, Trolox exhibited the highest superoxide radical scavenging activity. However, substantial inhibition of superoxide radicals was also observed for Lf and Lf-derived peptides. In most cases, Lf peptides showed higher scavenging activity than native Lf. The strongest activity was observed for trypsin- and chymotrypsin-derived Lf peptides (<50 kDa), while pepsin-derived peptides also demonstrated improved scavenging compared with Lf. Furthermore, peptides with molecular weights below 10 kDa displayed higher superoxide scavenging activity than the corresponding 10–50 kDa fractions for trypsin- and chymotrypsin-generated peptides. These results indicate that low-molecular-weight peptides (<10 kDa) contribute most to superoxide radical scavenging, consistent with previous reports [46,47].

#### 3.3.3. FRAP Assay

The antioxidant activity of Lf and Lf-derived peptides was further evaluated using the ferric reducing antioxidant power (FRAP) assay, which measures the ability of antioxidants to reduce Fe^3+^ to Fe^2+^. The resulting Fe^2+^ forms a blue complex with the TPTZ reagent, showing maximum absorbance at 593 nm [48]. FRAP values were calculated using a calibration curve prepared with FeSO_4_·7H_2_O and expressed relative to protein or peptide mass.

**Figure 4:**
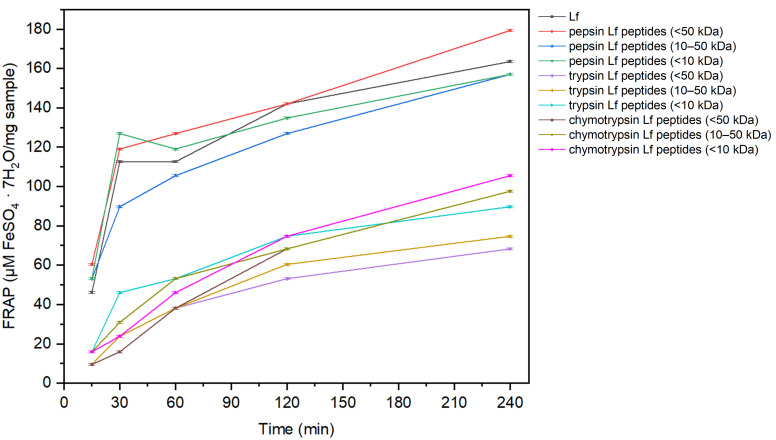
Antioxidant activity of Lf and pepsin, trypsin and chymotrypsin Lf peptides using the FRAP method as a function of time (minutes). The results represent the FRAP of Lf, Lf peptides (<50 kDa) and Lf peptides with a molecular weight of 10–50 kDa and <10 kDa. Data for Trolox are represented separately due to high FRAP values (Supplementary Figure S2).

Trolox showed substantially higher FRAP values than Lf and Lf-derived peptides; therefore, Trolox data are presented separately (see Supplementary Figure S2). Among the samples tested, pepsin-derived Lf peptides exhibited the highest reducing power compared with trypsin- and chymotrypsin-derived peptides (Figure 4). Notably, pepsin-derived peptides (<50 kDa) displayed higher reducing power than native Lf after 4 h of incubation. For trypsin- and chymotrypsin-derived peptides, the <10 kDa fractions showed the highest FRAP values, indicating that low-molecular-weight peptides contribute most significantly to ferric reducing capacity.

#### 3.3.4. Inhibition of Lipid Peroxidation

**Figure 5:**
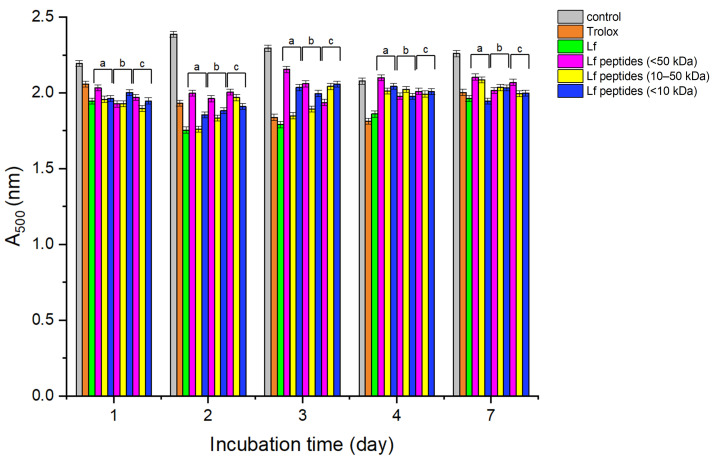
Inhibition of lipid peroxidation (%) of Lf and pepsin (a), trypsin (b) and chymotrypsin (c) Lf peptides as function of time (days). The results represent the inhibition of lipid peroxidation of Trolox, Lf, Lf peptides (<50 kDa) and Lf peptides with a molecular weight of 10–50 kDa and <10 kDa. Control contained no Lf and Lf peptides.

The ability of Lf and Lf-derived peptides to inhibit lipid peroxidation was evaluated using a linoleic acid oxidation model system. As shown in Figure 5, the control sample without Lf or Lf peptides showed the highest absorbance at 500 nm, indicating extensive lipid oxidation. Oxidation in the control reached a maximum after 2 days and then decreased, likely due to the formation of secondary degradation products [49].

Both native Lf and Lf-derived peptides significantly inhibited lipid peroxidation. Native Lf consistently showed strong inhibitory activity throughout the 7-day incubation period, with inhibition levels comparable to or greater than those of Trolox. Although Lf showed slightly better inhibition of lipid peroxidation, no significant differences were observed among Lf and peptides generated by pepsin, trypsin, or chymotrypsin on day 7, nor between peptide fractions of different molecular weights. These results indicate that both Lf and its derived peptides are effective inhibitors of lipid oxidation.

### 3.4. Mass Spectrometry

Hydrolysates produced by pepsin, trypsin, and chymotrypsin digestion of Lf were analysed using mass spectrometry. The identified peptide sequences and their corresponding molecular weights (Da) are shown in Table 2. As the entire Lf hydrolysates were subjected to mass spectrometric analysis without prior fractionation, only low-molecular-weight peptides could be reliably identified and sequenced. However, this limitation does not diminish the relevance of the analysis, as the antibacterial and antioxidant activities of Lf-derived peptides are generally attributed to peptides of low molecular weight. Therefore, the peptides identified in this study are considered representative and suitable for further discussion of the observed biological activities.

## 4. Discussion

In the present study, the food preservation potential of Lf and its hydrolysates was assessed by evaluating their antibacterial and antioxidant activity. The antibacterial activity of Lf and Lf hydrolysates was evaluated against four bacterial strains representing either foodborne pathogens or food spoilage microorganisms commonly associated with meat and dairy products. Our results demonstrated that Lf and Lf hydrolysates exhibited antibacterial activity against *E. coli*, *Li. monocytogenes*, and *Lb. sakei*, whereas little or no activity was observed against *S. epidermidis*.

*E. coli* O157:H7 [50] and *Li. monocytogenes* are well-recognized foodborne pathogens responsible for serious food poisoning. *Li. monocytogenes* is of particular concern due to its ability to persist in food-processing environments, contaminate ready-to-eat foods, and resist several antibiotics [51]. *S. epidermidis* is a common skin commensal frequently introduced into foods during processing, especially in meat and dairy products. Although it is rarely implicated in foodborne illness, it contributes to food spoilage through biofilm formation, particularly in ready-to-eat foods [52]. *Lb. sakei* is a lactic acid bacterium commonly found in fermented foods and plays an important role as a starter or protective culture in meat fermentation and preservation [53]. However, certain strains can produce exopolysaccharides in raw meat and fish products, leading to ropy slime formation and accelerated spoilage [54,55,56]. Consequently, both *S. epidermidis* and *Lb. sakei* are important targets for quality control in the food industry.

The antibacterial activity of Lf against *E. coli* observed in this study is most likely due to bactericidal mechanisms rather than iron sequestration, as previous studies have shown that Lf saturated with Fe^3+^ was not active against *E. coli* strains. In contrast, Lf demonstrated activity against the *Li. monocytogenes* strain even in its Fe^3+^-saturated form [35]. Further tests (not published) revealed a bacteriostatic effect on the *Li. monocytogenes* strain used in this study. Furthermore, the apo-form of Lf was used in this study without the addition of iron. While the bacteriostatic effect of Lf has been associated with its ability to bind iron and limit bacterial growth [57], the observed antibacterial activity against *Li. monocytogenes* can be attributed to direct interactions between Lf and bacterial cell envelope components. Previous studies have shown that Lf binds to lipopolysaccharides in the outer membrane of Gram-negative bacteria [58] and to lipoteichoic and teichoic acids in Gram-positive bacteria [59]. Such interactions destabilize the bacterial cell envelope, leading to membrane disruption and cell death.

Lf showed stronger antibacterial activity than the corresponding hydrolysates against all tested bacterial strains. However, hydrolysates derived from trypsin and chymotrypsin displayed notable inhibitory activity against *Li. monocytogenes*, and all three hydrolysates were particularly effective against *Lb. sakei*. According to existing literature, enzymatic hydrolysis (especially with pepsin) is often expected to enhance the antibacterial activity of Lf due to the release of potent peptides such as Lf-cin [19,23]. Furthermore, our experimental conditions also facilitated the formation of the antibacterial peptide Lf-cin (see Supplementary Figure S3). The lower antibacterial activity of the hydrolysates observed in this study may therefore be due to relatively low concentrations of highly active antibacterial peptides within the complex hydrolysate mixtures, as also reported by Conesa et al. (2008) [60]. This is also supported by the low concentration of Lf-cin in our pepsin-mediated hydrolysate (see Supplementary Figure S3). Furthermore, enhanced antibacterial effects are more commonly observed for isolated Lf-derived peptides, while whole hydrolysates rarely exhibit stronger activity than native Lf [19,20,61]. Despite this, the Lf hydrolysates still demonstrated measurable antibacterial activity against both foodborne pathogens and spoilage microorganisms, indicating their potential as natural antimicrobial agents in the food industry.

Fractionation of Lf hydrolysates into different molecular weight ranges, combined with mass spectrometry analysis, provided further insight into the antibacterial properties of Lf-derived peptides. The antibacterial activity of the hydrolysates appeared to depend more on peptide composition than on molecular weight alone. Separation into high- (10–50 kDa) and low-molecular-weight (<10 kDa) fractions did not enhance antibacterial activity compared with the unfractionated hydrolysates, which consistently showed the strongest effects. This suggests that synergistic interactions among multiple peptides, as well as the presence of residual native Lf, contribute to the overall antibacterial activity. From a practical perspective, these findings indicate that whole hydrolysates may be more effective and economically viable for food applications than purified peptide fractions, as they avoid additional processing steps and retain antibacterial efficacy. Antimicrobial peptides are typically characterized by short amino acid sequences (10–50 residues), a net positive charge, and a high proportion of hydrophobic residues [62]. Mass spectrometry analysis confirmed the presence of peptides with these features in the Lf hydrolysates (Section 3.4), supporting their role in the observed antibacterial effects.

In addition to antibacterial activity, the antioxidant properties of Lf and Lf peptides were evaluated using four complementary assays targeting a broad range of free radicals and oxidative mechanisms. By integrating the results on the antioxidant activity of Lf and Lf-derived peptides, the present study showed that both Lf and its peptides exhibited a relatively low antioxidant capacity compared to Trolox. Similar findings have been reported in previous studies, where the antioxidant activity of Lf was lower than that of strong antioxidants such as vitamin C, butylated hydroxytoluene (BHT), or ascorbic acid [31,63]. These studies also demonstrated that the antioxidant activity of Lf is concentration-dependent. In the present study, antioxidant activity was evaluated at a single concentration (0.25 mg/mL), as higher concentrations led to protein aggregation (in the DPPH assay). Consistent with previous reports, our results confirm that bioactive peptides generally exhibit lower antioxidant activity than potent reference antioxidants [39,49]. Nevertheless, both Lf and Lf-derived peptides showed measurable antioxidant activity in the DPPH radical scavenging, superoxide radical scavenging, and FRAP reducing power assays. The most pronounced antioxidant effect was observed in the lipid peroxidation inhibition assay, where native Lf and its peptides demonstrated antioxidant activity comparable to that of Trolox after a 7-day evaluation period. These findings suggest that Lf and its derived peptides can act as natural antioxidants, particularly in inhibiting lipid oxidation, which is a major contributor to quality deterioration and reduced shelf life in lipid-containing foods. In addition to their relevance for food preservation, Lf-derived peptides may also exert antioxidant effects in vivo, as they can be naturally generated during gastrointestinal digestion. Their ability to modulate reactive oxygen species in the gut may therefore contribute to reducing oxidative stress at the cellular level.

In the DPPH radical scavenging assay, Lf and its derived peptides showed comparable antioxidant activity, whereas in the superoxide radical scavenging and FRAP assays, Lf-derived peptides exhibited stronger antioxidant activity than Lf. Previous studies have shown that the antioxidant activity of bioactive peptides is influenced by several factors, including molecular weight, amino acid composition, sequence, and hydrophobicity [34]. By fractionating Lf hydrolysates into higher- (10–50 kDa) and lower-molecular-weight (<10 kDa) peptide fractions and performing mass spectrometry analysis, we gained further insight into the mechanisms underlying the antioxidant activity of Lf-derived peptides.

Across all four antioxidant assays used in this study, low-molecular-weight Lf peptides (<10 kDa) consistently showed stronger or, in the case of lipid peroxidation inhibition, comparable antioxidant activity relative to higher-molecular-weight fractions, regardless of the enzyme used for hydrolysis. These findings indicate that small peptides contribute most significantly to the antioxidant activity of Lf-derived peptides and are consistent with previous reports on food-derived bioactive peptides [46,64].

The amino acid composition in sequences of Lf-derived peptides (<50 kDa) (Table 2) confirmed that antioxidant activity was closely linked to peptide composition. The high abundance of hydrophobic residues, particularly alanine (Ala), valine (Val), leucine (Leu), phenylalanine (Phe), tyrosine (Tyr), proline (Pro), and glycine (Gly), in these peptides likely contributed to their ability to inhibit both DPPH radicals and lipid oxidation, through their ability of direct electron transfer (i.e., hydrogen donors) [65,66]. The DPPH radical (2,2-diphenyl-1-picrylhydrazyl) is a stable free radical neutralized by hydrogen atom donation from antioxidants, resulting in the formation of a stable non-radical species (DPPH-H) [67]. Therefore, hydrogen-donating capacity is a key mechanism underlying DPPH radical scavenging. In addition to hydrophobic residues, the relatively high content of arginine (Arg) and lysine (Lys) in Lf peptides (<50 kDa) may have contributed to DPPH radical inhibition, as moderate scavenging activity has previously been reported for these amino acids [68]. Hydrophobic amino acid residues also play a crucial role in inhibiting lipid peroxidation. These residues can act as hydrogen donors to quench lipid radicals, while their hydrophobic nature enhances peptide affinity for lipid phases. This property likely facilitates interactions with lipophilic reactive oxygen species and polyunsaturated fatty acids, thereby improving the efficiency of lipid oxidation inhibition [66].

Superoxide radical scavenging is primarily associated with the ability of compounds to donate electrons or hydrogen atoms; therefore, amino acid residues possessing these properties are likely to play a key role in the antioxidant activity of peptide sequences. The superoxide scavenging activity observed for Lf-derived peptides can be attributed to their high content of leucine (Leu) and lysine (Lys), as well as acidic amino acids (aspartic acid and glutamic acid) and aromatic residues such as phenylalanine (Phe) and tyrosine (Tyr). These amino acids were particularly abundant in trypsin- and chymotrypsin-derived Lf peptides (<50 kDa), which is consistent with their higher superoxide radical scavenging activity. Across all Lf peptide fractions (<50 kDa), Leu was present in the highest proportion, suggesting that this residue plays a significant role in the inhibition of superoxide radicals.

Pepsin-derived Lf peptides (<50 kDa), as well as low-molecular-weight trypsin- and chymotrypsin-derived peptides (<10 kDa), exhibited the highest antioxidant activity in the FRAP assay. In these samples, the content of cysteine (Cys) and methionine (Met), amino acids commonly associated with metal-chelating and antioxidant properties [69], was relatively low. This indicates that the observed FRAP activity of Lf-derived peptides cannot be primarily attributed to sulphur-containing residues. Instead, these peptides were enriched in other electron-donating amino acids, including aspartic acid (Asp), glutamic acid (Glu), proline (Pro), and glycine (Gly), suggesting that these residues play a major role in the ferric reducing activity. Interestingly, although trypsin- and chymotrypsin-derived peptides (<50 kDa) contained higher overall levels of these electron-donating amino acids than pepsin-derived peptides, their FRAP activity was lower than expected. This apparent discrepancy prompted further consideration of peptide composition beyond simple amino acid abundance. Previous studies have shown that certain residues, particularly positively charged and aromatic amino acids, can negatively influence FRAP measurements [47,70]. These residues were more abundant in trypsin- and chymotrypsin-derived peptides, which likely reduced their overall ferric reducing capacity. Consequently, the higher FRAP activity observed for pepsin-derived Lf peptides is best explained by a favourable balance between low-molecular-weight peptides and electron-donating amino acid residues, combined with a lower proportion of residues that inhibit FRAP activity.

## 5. Conclusions

This study demonstrates that both native Lf and its enzymatic hydrolysates possess biologically relevant antibacterial and antioxidant activities applicable to food preservation. Lf exhibited the strongest antibacterial activity against the tested foodborne pathogens and spoilage microorganisms, while Lf hydrolysates, particularly those produced with trypsin and chymotrypsin, retained notable inhibitory effects against *Li. monocytogenes* and *Lb. sakei*. Importantly, this work provides the first evidence of antibacterial activity of Lf and its hydrolysates against *Lb. sakei*, a significant spoilage organism in meat products.

The antibacterial efficacy of Lf-derived peptides depended more on peptide composition and synergistic interactions within whole hydrolysates than on molecular weight alone, as fractionated peptides showed reduced or no antibacterial activity. These findings highlight the importance of maintaining the complex peptide mixture, together with residual native Lf, to achieve optimal antimicrobial effects. Antioxidant evaluation revealed that although Lf and its peptides exhibited lower overall activity than a strong reference antioxidant, they were effective inhibitors of lipid peroxidation, with Lf demonstrating particularly strong and sustained activity. Low-molecular-weight peptides contributed most to radical scavenging and reducing power, as supported by mass spectrometry analysis, which revealed peptides enriched in hydrophobic and electron-donating amino acid residues.

Overall, these results support the potential use of Lf and its enzymatic hydrolysates as natural antimicrobial and antioxidant agents in food systems to enhance safety and extend shelf life. From a practical perspective, using whole hydrolysates, particularly those derived from whey as a dairy industry by-product, offers a more effective and economically viable alternative to purified peptide fractions, providing a sustainable strategy for industrial-scale food preservation.

## Figures and Tables

**Table 1:** Antibacterial activity of Lf and Lf peptides obtained by enzymatic hydrolysis of pepsin, trypsin, and chymotrypsin on the growth of indicator strains. MIC value—the lowest concentration (in mg/mL) at which the sample inhibits the growth of the test strain by at least 50%. We estimated the growth as OD_630_.

Sample	*Latilactobacillus sakei* ATCC 15522	*Listeria* *monocytogenes* IM 221	*Staphylococcus epidermidis* ATCC 14990	*Escherichia coli* IM 219
Lf	0.01	0.6	4.8	2.4
Pepsin Lf hydrolysate (unfractioned)	0.01	2.4	>19.25	>19.25
Pepsin Lf peptides (10–50 kDa fraction)	0.6	>19.25	>19.25	>19.25
Pepsin Lf peptides (<10 kDa fraction)	>19.25	>19.25	>19.25	>19.25
Trypsin Lf hydrolysate (unfractionated)	0.02	0.6	>19.25	4.8
Trypsin Lf peptides (10–50 kDa fraction)	1.2	>19.25	>19.25	>19.25
Trypsin Lf peptides (<10 kDa fraction)	>19.25	>19.25	>19.25	>19.25
Chymotrypsin Lf hydrolysate (unfractioned)	0.02	1.2	19.25	4.8
Chymotrypsin Lf peptides (10–50 kDa fraction)	1.2	>19.25	>19.25	>19.25
Chymotrypsin Lf peptides (<10 kDa fraction)	>19.25	>19.25	>19.25	>19.25

**Table 2:** Sequence of the peptides obtained by the enzymatic hydrolysis with pepsin, trypsin, and chymotrypsin with their corresponding molecular weight (kDa).

Pepsin Hydrolysate		Trypsin Hydrolysate		Chymotrypsin Hydrolysate	
Peptide Sequence	Peptide Molecular Weight (Da)	Peptide Sequence	Peptide Molecular Weight (Da)	Peptide Sequence	Peptide Molecular Weight (Da)
AVAPNHAVVSRSDRAAHVKQVL	2325.29	AFALECIR	979.50	AEDVGDVAF	922.42
DCVLRPTEGYL	1322.64	CGLVPVLAENR	1227.65	AKNLNREDF	1106.56
DGGYIYTAGKCGLVPVL	1782.91	CGLVPVLAENRK	1355.75	AVAVVKKANEGLTW	1485.84
ENLPEKADRDQYEL	1719.82	CLQDGAGDVAFVK	1379.66	AVAVVKKANEGLTWNSL	1801.97
ENLPEKADRDQYELL	1832.90	DLLFKDSALGFLR	1494.83	AVAVVKKGSNF	1119.65
EPLQGAVAKF	1059.58	DSALGFLR	878.47	CAGDDQGL	835.33
FGSPPGQRDL	1073.54	ECHLAQVPSHAVVAR	1673.85	CAVGPEEQKKCQQW	1747.79
FGSPPGQRDLL	1186.62	ETTVFENLPEK	1306.65	CTISQPEW	1020.45
FKDSALGF	884.45	ETTVFENLPEKADR	1648.82	CTISQPEWF	1167.51
FKSETKNLL	1079.61	GSNFQLDQLQGR	1362.68	DCVLRPTEGY	1209.56
GRSAGWIIPMGIL	1370.76	GSNFQLDQLQGRK	1490.77	DGTRKPVTEAQSCHL	1698.82
GSPPGQRDL	926.47	HSSLDCVLRPTEGYLAVAVVK	2314.22	ENLPEKADRDQY	1477.69
GSPPGQRDLL	1039.55	KANEGLTWNSLK	1360.72	ENTNGESTADW	1223.48
KDSALGFL	850.47	KGSNFQLDQLQGR	1490.77	FKDSALGF	884.45
KGEADALNL	930.49	KPVTEAQSCHLAVAPNHAVVSR	2371.23	GGRPTYEEY	1071.47
KKCSTSPLL	1033.57	LGAPSITCVR	1073.58	GGRPTYEEYL	1184.56
KSETKNLL	932.54	LLCLDGTR	947.50	GSPPGQRDL	926.47
LRIPSKVDSAL	1198.72	LRPVAAEIYGTK	1317.75	GSPPGQRDLL	1039.55
PEKADRDQYELL	1477.72	NLLFNDNTECLAK	1551.75	GSPPGQRDLLF	1186.62
QLFGSPPGQRDL	1314.68	NLNREDFR	1063.53	GTKESPQTHYY	1310.60
SWTESLEPLQGAVAKF	1762.90	NLRETAEEVK	1188.62	IIPMGILRPY	1172.69
VLKGEADAL	915.51	QVLLHQQALFGK	1381.80	KCLQDGAGDVAF	1280.59
VLKGEADALNL	1142.64	SFQLFGSPPGQR	1322.64	KDSALGFL	850.47
ENLPEKADRDQYEL	1719.82	SVDGKEDLIWK	1289.67	KGEADALNLDGGY	1322.62
EPLQGAVAKF	1059.58	WCTISQPEWFK	1481.69	KLRPVAAEIY	1159.68
FGSPPGQRDL	1073.54	YYGYTGAFR	1097.51	KNLRETAEEVKARY	1706.92
GSPPGQRDL	926.47	AFALECIR	979.50	LRIPSKVDSAL	1198.72
GSPPGQRDLL	1039.55	CGLVPVLAENR	1227.65	LRIPSKVDSALY	1361.78
KGEADALNL	930.49	CGLVPVLAENRK	1355.75	NNSRAPVDAF	1090.53
KSETKNLL	932.54	CLQDGAGDVAFVK	1379.66	QGAVAKFF	867.47
LRIPSKVDSAL	1198.72	DSALGFLR	878.47	RCLAEDVGDVAF	1351.63
VLKGEADAL	915.51	EPYFGYSGAFK	1265.58	RETAEEVKARY	1351.70
		ESPQTHYYAVAVVK	1591.81	RIPSKVDSAL	1085.63
		ETAEEVKAR	1032.53	RIPSKVDSALY	1248.69
		ETTVFENLPEK	1306.65	RPVAAEIY	918.50
		ETTVFENLPEKADR	1648.82	SQSCAPGADPKSRL	1473.71
		GSNFQLDQLQGR	1362.68	SWTESLEPL	1061.52
		GSNFQLDQLQGRK	1490.77	TAGKCGLVPVL	1114.63
		KANEGLTWNSLK	1360.72	TESLEPLQGAVAKF	1489.79
		KGSNFQLDQLQGR	1490.77	VLKGEADAL	915.51
		KLGAPSITCVR	1201.67	VLKGEADALNL	1142.64
		LGAPSITCVR	1073.58	YAVAVVKKGSNF	1282.72
		LLCLDGTR	947.50	AEDVGDVAF	922.42
		LRPVAAEIYGTK	1317.75	AKNLNREDF	1106.56
		NLNREDFR	1063.53	AVAVVKKGSNF	1119.65
		NLRETAEEVK	1188.62	CTISQPEWF	1167.51
		QVLLHQQALFGK	1381.80	ENLPEKADRDQY	1477.69
		SFQLFGSPPGQR	1320.67	ENTNGESTADW	1223.48
		SVDGKEDLIWK	1289.67	FKDSALGF	884.45
		WCTISQPEWFK	1481.69	GGRPTYEEY	1071.47
		YYGYTGAFR	1097.51	GGRPTYEEYL	1184.56
				GSPPGQRDL	926.47
				GSPPGQRDLL	1039.55
				GSPPGQRDLLF	1186.62
				GTKESPQTHY	1147.54
				GTKESPQTHYY	1310.60
				KCLQDGAGDVAF	1280.59
				KDSALGFL	850.47
				KGEADALNLDGGY	1322.62
				KLRPVAAEIY	1159.68
				KSETKNLLF	1079.61
				NNSRAPVDAF	1090.53
				QDGAGDVAF	879.38
				QGAVAKFF	867.47
				RETAEEVKARY	1351.70
				RIPSKVDSAL	1085.63
				RIPSKVDSALY	1248.69
				RPVAAEIY	918.50
				SWTESLEPL	1061.52
				TAGKCGLVPVL	1114.63
				TESLEPLQGAVAKF	1489.79
				VLKGEADAL	915.51
				YAVAVVKKGSNF	1282.72

## Data Availability

The original contributions presented in this study are included in the article/Supplementary Materials. Further inquiries can be directed to the corresponding author.

## Supplementary Materials

The following supporting information can be downloaded at https://www.mdpi.com/article/10.3390/foods15061052/s1: Figure S1: SDS-PAGE results of Lf enzymatic hydrolysis with 3% (*w*/*w*) and 6% (*w*/*w*) trypsin concentrations based on the mass of Lf. Both enzymatic hydrolyses were performed in the presence and absence of 20 mM CaCl_2_ for 4 h at 37 °C in a water bath.; Figure S2: Antioxidant activity of Trolox measured with the FRAP method; Figure S3: HPLC analysis of pepsin Lf hydrolysate and Lf-cin.

## Abbreviations

The following abbreviations are used in this manuscript:

Lf	Lactoferrin
FDA	Food and Drug Administration
EFSA	European Food Safety Authority
Lf-cin	Lactoferricin
MIC	Minimal Inhibitory Concentration
DPPH	2,2-diphenyl-1-picrylhydrazyl
TPTZ	2,4,6-Tris(2-pyridyl)-s-triazine
FRAP	Ferric Reducing Antioxidant Power
