# Supplementary material for: Lactoferrin and Its Enzymatic Hydrolysates as Natural Antimicrobial and Antioxidant Agents for Food Preservation
Source: Foods. 2026 Mar 17;15(6):1052. doi: 10.3390/foods15061052 (PMC13026072; doi:10.3390/foods15061052)
Supplement: Supplementary file 1 [file foods-15-01052-s001.zip › foods-4158873-supplementary.pdf]

Supplementary material

Figure S1. SDS-PAGE results of Lf enzymatic hydrolysis with 3% (*w*/*w*) and 6% (*w*/*w*) trypsin concentrations based on the mass of Lf. Both enzymatic hydrolyses were performed in the presence and absence of 20 mM $CaCl_2$ for 4 h at 37 °C in a water bath.

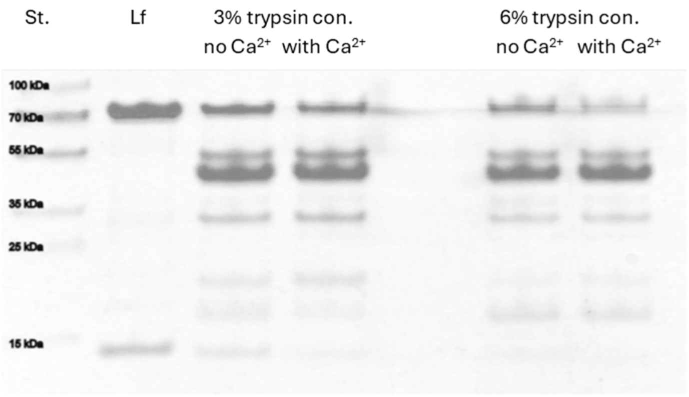

Figure S2: Antioxidant activity of Trolox measured with the FRAP method.

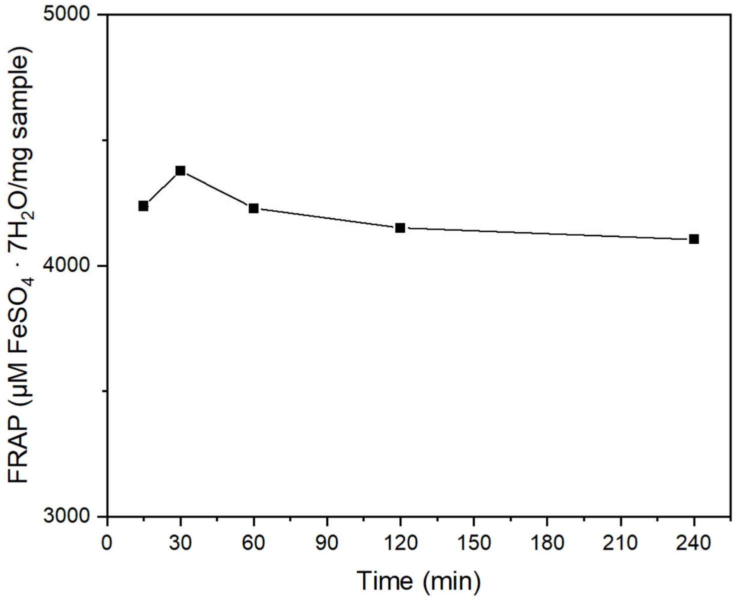

Figure S3: HPLC analysis of pepsin Lf hydrolysate and Lf-cin.

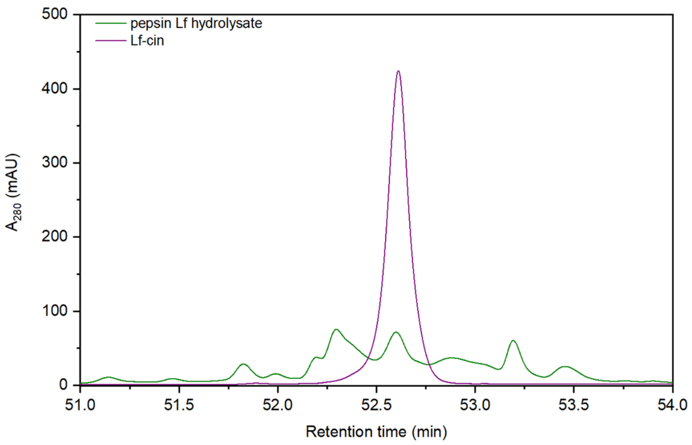

Method of HPLC analysis: Lf hydrolysate obtained by enzymatic pepsin hydrolysis and commercial Lf-cin were analysed by high-resolution liquid chromatography on an Infinity 1260 II LC system. For the HPLC analysis, a C12 chromatographic column (Jupiter® 4 µm Proteo 90 A) was used. Solutions A and B were prepared and used as mobile phases. Solution A contained 0.09% (*v*/*v*) trichloroacetic acid (TCA) dissolved in milliQ water, while solution B contained 0.01% (*v*/*v*) TCA and 90% (*v*/*v*) acetonitrile in milliQ. Pepsin Lf peptides and Lf-cin were dissolved in HPLC analysis buffer, at a concentration of 5 mg/mL, which consisted of 95% (*v*/*v*) solution A and 5% (*v*/*v*) solution B. A linear gradient of solvent B and A from 10% to 90% in 71 minutes at 25 °C and a flow rate of 0.8 mL/min. The absorbance was measured at 280 nm.
